# Self-Sealing Process Evaluation Method Using Ultrasound Technique in Cement Composites with Mineral Additives

**DOI:** 10.3390/ma13153336

**Published:** 2020-07-27

**Authors:** Kamil Tomczak, Jacek Jakubowski, Łukasz Kotwica

**Affiliations:** 1 Department of Geomechanics, Civil Engineering and Geotechnics, AGH University of Science and Technology, 30-059 Krakow, Poland; ktomczak@agh.edu.pl; 2 Department of Building Materials Technology, AGH University of Science and Technology, 30-059 Krakow, Poland; lkotwica@agh.edu.pl

**Keywords:** self-healing, self-sealing, cementitious material, supplementary cementitious materials, crack in concrete, non-destructive testing, ultrasound techniques, ultrasonic pulse velocity

## Abstract

The self-sealing process, associated with chemical and microstructural changes inside damaged cement-based composites, leads to the recovery of the original material integrity. Assessing the magnitude of internal changes in samples using non-destructive techniques to capture only the self-sealing effects is difficult. The challenge is evaluating the differences between subsequent observations in time and between samples with different properties. This paper proposes a new approach to the use of an ultrasonic technique for self-sealing investigation. The method allows the quantification of material changes strictly related to self-sealing processes, excluding changes caused by the naturally progressing hydration of binders. The applied ultrasonic pulse velocity (UPV) data processing procedure allows the investigation of material changes inside and near the cracks, the effects of stimulating the self-sealing of cement composites with mineral additives, and the assessment of changes over time. An important aspect of the method is the sample preparation procedure and testing conditions that reduce the impact of moisture content on the UPV measurements. New parameters allowing the quantitative characterization of the self-sealing process are proposed. The method was evaluated using cement mortars modified with siliceous fly ash with induced cracks 0 to 750 µm wide, which were then cured in water for 152 days. The maximum degree of effective crack filling as a result of autogenous self-sealing in the tested mortars was determined to range from 33% to 57%. Observations of the microstructure of the crack surface confirmed that apart from the volume of the newly formed products, the density of these products may have a key impact on the ultrasonic measurements of the self-sealing performance. The studies were supplemented by the examination of the compression strength of mortars, mortar sample scanning and computer image processing, and observations using an optical microscope and scanning electron microscope with energy dispersive spectroscopy.

## 1. Introduction

Cement-based self-healing composites are an example of a new generation of materials [1]. Although the topic of self-healing as described by van Breugel [2] dates back to the 19th century, the most intense period of development of research methods and research in this field has occurred in the last two decades. To date, researchers have focused on the possibility of increasing the autogenous self-healing capacity of cement composites by introducing mineral additives [3,4,5,6] or the development of self-healing engineering techniques based on bacteria [7,8,9] or adhesives [10]. Some of the activities focused on reducing crack width in engineered cementitious composite (ECC) [11] or ensuring internal curing using superabsorbent polymers [12]. In self-healing tests, the ability of composites to restore the original stiffness or strength lost due to material degradation is considered. However, in some studies, the focus was only on the ability to self-seal cracks with the newly created material and the effectiveness of this process in restoring the original material permeability [13]. This aspect is particularly important when the cement composite provides passive protection for reinforcing steel bars against the influence of the external environment.

**Figure 1:**
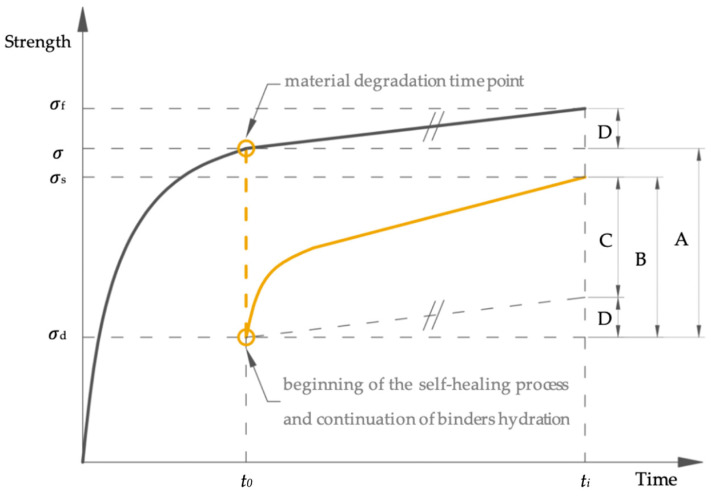
Changes in cement-based composite strength due to undisturbed hydration of the binder (black line) and self-healing after degradation of material (yellow line). Note: A, the strength lost due to material degradation at time point *t_0_*; B, the strength gain during self-healing; C, the strength gain derived exclusively from self-healing; D, the strength gain due to further hydration of binders; *t_i_*, any time point of observation; σ_f_, final strength; σ, strength at the moment of material degradation; σ_d_, strength after material degradation; σ_s_, strength after self-healing; based on [14].

During the research on the self-healing of cement-based composites, when assessing the scale of the changes caused by autogenous self-healing in particular, the change in material properties over time through the progressive hydration of binders [14,15,16] should be considered. Figure 1 presents a general diagram of changes in cement-based composite strength during maturation and the restoration of strength during self-healing (at any time point *t*_i_ of the research) that was lost due to material degradation (at time point *t*_0_). The black solid line shows the uninterrupted progress of the strength increase over time. The yellow dashed line shows the loss of strength as a result of damage at time point *t_0_* (value “A”), whereas the yellow solid line describes further changes in strength during subsequent self-healing up to time point *t_i_* (value “B”). The dynamics of the maturing and self-healing process vary over time [17] and primarily depend on the activity of the binders used and the curing conditions, as demonstrated by the non-linear course of strength development. Some of the changes registered during the tests as self-healing effects result from the natural course of further hydration of the binder (value “D”). The strength gain due to pure self-healing (value “C”) is the difference between the total strength gain during self-healing (value “B”) and the strength gain due to further hydration of the binder (value “D”) [14].

Non-destructive ultrasonic techniques are commonly used to evaluate the mechanical properties of cement-based composites. The incorporation of an ultrasonic technique allows the tracing of the strength evolution of the samples, which allows the analysis of the changes depicted in Figure 1. This solution allows the observation of the changes in the properties of the material without interrupting or interfering with the examined processes.

In general, the ultrasonic techniques used for material characterization differ in how the wave is induced, its type and properties, the phenomena accompanying the propagation of waves in the material (such as reflection, scattering, or absorption by material particles), and the equipment used. In the field of research on the properties of cement-based composites, including self-healing or self-sealing, techniques based on ultrasonic pulse velocity (UPV) [18,19,20,21,22,23,24,25], acoustic emission [26,27,28], surface-wave transmission [29], diffuse ultrasound [30], or coda wave interferometry are used [31].

The ultrasonic testing techniques most commonly used for the examination of the properties of cementitious composites are based on the measurement of ultrasonic wave passage time. The main variations in the application of this technique are related to the wave frequency (usually from 20 to 100 kHz) and the direction of wave generation in relation to the object under investigation, i.e., the surfaces to which transmitting and receiving transducers are applied. In this respect, the three types of UPV tests are direct transmission (cross probing), semi-direct transmission, and indirect transmission (surface probing) [19]. Camara et al. [20] used UPV tests in cylindrical concrete samples in which defects were induced by compressive stress and subsequent self-healing under conditions of cyclic immersion in a lime-saturated solution and drying at 22 ± 3 °C, a relative humidity of 75.4% ± 6.6%, and a carbon dioxide concentration of 0.75% ± 0.13%. Zhong and Yao [21] used ultrasonic techniques in combination with mechanical tests to prove that a certain threshold of material damage degree exists, below which the ability of concrete to self-heal increases with the degree of damage and above which this ability decreases with the degree of damage. Zhu et al. [22] used the transition time of ultrasonic waves as a measure of self-healing in engineered cementitious composite (ECC) samples subjected to different levels of deformation and subsequent wetting and drying cycles. Cappallesso et al. [23] studied changes in the UPV associated with the formation of material defects after wetting and drying cycles on rectangular concrete samples with different water-to-binder ratios. The samples in the cited studies cracked after 3 and 28 days of hydration [23].

The approaches used in the tests referred to in the previous paragraph may raise some doubts, since the measurements of UPV were conducted on samples with different moisture contents, as they were initially immersed in water. From theoretical considerations, which were confirmed during preliminary tests and in previous studies [32], the chemically unbound water remaining in defects and pores in partially or fully saturated cement-based composites increases the UPV compared to the cement-based composite at the same stage of hydration but in an air-dry state or after drying in an oven. The variations in moisture content affect the accurate determination of UPV changes and thus complicate the assessment of the course of the self-healing process. Güneyli et al. [33] determined that a 1% water content in concrete samples increases the UPV by 160 m/s, whereas increasing the temperature of samples by 10 °C increases the UPV by 34 m/s. Huang et al. [24] conducted self-healing tests on fiber-reinforced concrete with the addition of ground rubber, heated the samples at 80 °C for one hour, and then cooled them to room temperature to eliminate the impact of water on the results of the UPV tests. Multiple high-temperature dryings of a sample change the hydration process course; therefore, this approach cannot be used if multiple measurements of a particular sample are necessary.

In response to the deficiencies of the ultrasonic testing methods used for the examination of the self-sealing process in cement composites, a new approach to sample preparation and testing conditions is proposed here. This approach, in particular, considers actions to reduce the variations in UPV test results due to differences in the moisture contents of the materials tested. Despite the widespread use of ultrasonic techniques, sensitivity to testing conditions and the accuracy of measurements remain current problems [19]. Some other technical issues and remarks related to sample preparation, the introduction of material defects, and cracks’ geometry analysis were also considered [34,35,36,37]. The proposed method, including self-sealing process evaluation, the experimental groups’ composition, ultrasound measurements, and the data analysis, is presented here through tests of the autogenous self-sealing of cracks in polypropylene-fiber-reinforced mortars modified with siliceous fly ash. To facilitate a correct conclusion based on the results, these studies were supplemented by studies on the relationship between the compression strength of mortar and UPV, and observations using an optical microscope and scanning electron microscope (SEM) with energy dispersive spectroscopy (EDS).

## 2. Materials and Testing Procedures

A total of 36 rectangular 40 × 40 × 160 mm mortar samples were tested. The material changes during the mechanical induction of cracks after 28 days of maturing and subsequent self-sealing for a further 152 days of curing on samples composed of 6 mortars were investigated. The mortars had a constant total binder mass. Portland cement CEM I 42.5R was partially substituted by siliceous fly ash from bituminous coal combustion in a pulverized coal-fired boiler. The amounts of fly ash introduced were 0%, 5%, and 20% of the total binder mass. Two water-to-binder ratios (w/b), 0.3 and 0.5, were used. The chemical compositions of the cement and siliceous fly ash are presented in Table 1.

CEN-standard sand according to PN-EN 196-1 [38] was used as a fine aggregate. CHRYSO_®_ Optima 185 superplasticizer (based on modified polycarboxylates and phosphonates) was used for adjusting the workability of the fresh mortar mixes. The superplasticizer dosage was chosen to ensure comparable workability during mixing and no significant air content difference in the fresh mortars (which was measured using the gravimetric method). Astra Polyex Mesh 2000_®_ high-modulus polypropylene fibers, 12 mm long (dosing = 2% of mortar volume), were introduced to the mortars to increase the control of the deformation of the samples during crack induction and to ensure the geometric stability of the samples during the later stages of the experiment. The mix proportions of the investigated mortars are shown in Table 2. *Mp_q* denotes mortar with *p*% substitution of cement with siliceous fly ash and w/b = *q*.

**Figure 2:**
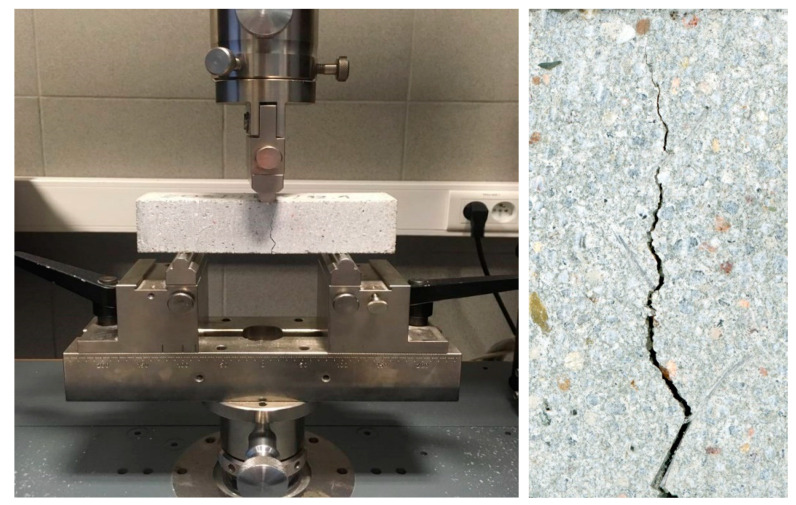
Sample immediately after the induction of the defect by three-point bending, and a typical image of the single crack with variable width (side wall of the sample, parallel to the force direction).

Samples were cast in steel molds, covered with plastic foil, and stored for 24 h at 20 ± 2 °C and ≥90% relative humidity (RH). After demolding, the samples were matured for 27 days at 20 ± 2 °C and ≥90% RH in sealed containers. A layer of 40 to 100 µm-thick cement paste was ground from the side surfaces of the samples before the next part of the study using a stationary grinding machine. This action was aimed at reducing the edge effect of the newly formed material accumulating in the crack area at the outer surface of the sample. The samples were also dried under the conditions described in Section 3. Then, 3 samples for each mortar were used as control samples. The remaining 3 samples for each mortar were loaded to induce a macrodefect in the form of a single crack. For this purpose, the samples were subjected to three-point bending tests under conditions of displacement velocity control at 1 mm/min and a preset deformation limit in the form of deflection of 1.75%. The size of the introduced deformations was selected based on preliminary tests so that cracks with a variable width from 0 to a maximum of 750 µm formed on the sides of the samples. A high-precision Zwick Roell Xforce P bending test machine (ZwickRoell GmbH & Co. KG, Ulm, Germany) was used, with a load range of up to 20 kN, and equipment to perform three-point bend tests with 10 mm diameter supports and a load stamp (Figure 2).

**Figure 3:**
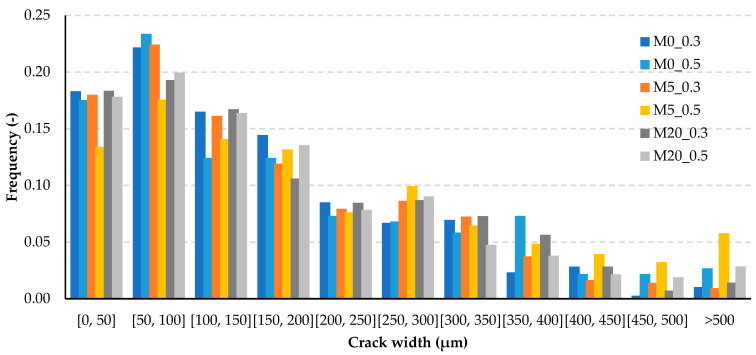
Histogram of the initial cracks’ width (directly after cracking) based on the multiple measurements along the cracks with the use of computer processing and analysis of high-resolution samples surface images.

The procedure for inducing cracks was designed to introduce a single crack into each of the samples tested. The goal was to have the width of the crack similar for all samples. To evaluate the crack geometry, the side surfaces of the samples were scanned using a high resolution Epson V600 Photo 6400 dpi scanner (Seiko Epson Corporation, Tokyo, Japan). The initial crack width was measured using a method previously described [34] using computer image processing and analysis. Multiple measurements were recorded automatically and independently of the operator along the cracks. For each of the mortars, 418 ± 16 measurements on average were taken on a total of 6 side surfaces of the 3 samples (2 side surfaces for each of the samples). Each measurement is the width of the crack in a particular place. The results are shown in Figure 3 as histograms of the width distribution along the crack length.

Scans of the samples’ surfaces were also collected after crack induction, during the curing, at the ultrasonic test time points (Section 3). The obtained cracks’ width measurements based on the abovementioned method of computer image processing and analysis were used to investigate the relationship between the reduction of crack width at the outer surfaces of the samples and changes occurring during the self-sealing process inside the samples (Section 5).

The histograms for all the mortars were characterized by a similar distribution of crack widths arranged in characteristic intervals of 50 µm. For all the mortars, nearly 95% of the cracks’ widths were below 500 µm, with nearly half of the cases below 150 µm.

The research plan also provided for tests of the changes in the compressive strength of the mortars after 28, 56, 90, 120, 150, and 180 days of maturation. For this purpose, a total of 108 rectangular 25 × 25 × 100 mm mortar samples were prepared (3 samples for each of the 6 mortars tested at 6 time points). They were made from the mortars with the formulas provided in Table 2 but without polypropylene fibers. The research aimed to test the compressive strength as a measure of binder activity (properties of cementitious matrix), and the diffuse reinforcement would disturb this assessment by modifying the tensile strength of the composite.

The microstructure of the composite fractures was observed on an ultra-high resolution scanning electron microscope, Nova NanoSEM 200, produced by FEI Europe Company (Eindhoven, The Netherlands), with an EDAX EDS analyzer. The surface of the fractures was observed in secondary electron mode (SE) at an accelerating voltage of 18 kV and magnification from 350× to 10,000×.

## 3. UPV Measurements During the Self-Sealing Process

**Figure 4:**
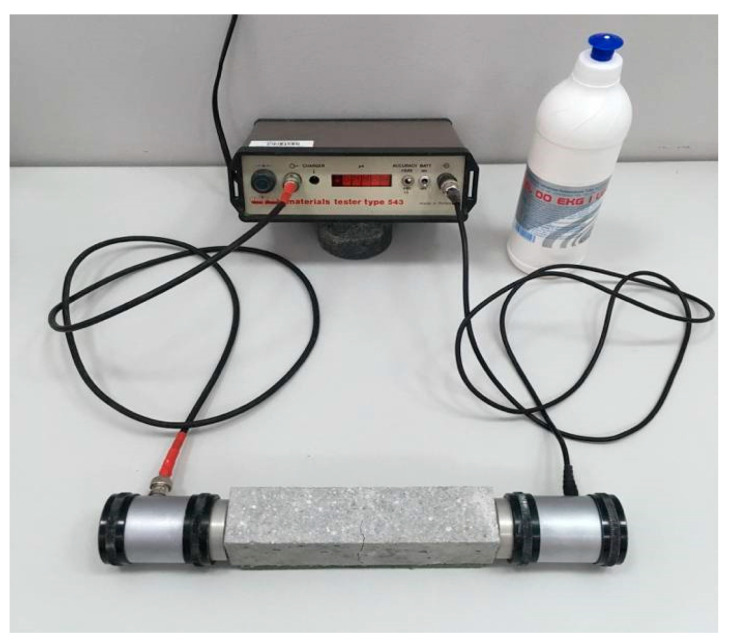
Test stand for testing the ultrasonic pulse velocity by direct transmission in rectangular mortar samples.

Non-destructive UPV tests were used to evaluate changes within the material due to the induced crack, as well as those caused by the potential self-sealing process. The time of ultrasonic wave passage through the sample was measured directly before and after the induction of cracks on the 28th day of matuation (at time point *t*_0_), and after *t_i_* = 1, 3, 7, 14, 28, 62, 92, 122, and 152 days of curing. Thus, UPV was measured at a total of 11 time points. Changes in UPV resulting from further hydration of the binders were tested using control samples without the intentionally introduced defects at the respective time points. These measurements were recorded using an ultrasonic defectoscope Unipan type 543 material tester (Unipan, Warsaw, Poland), with transmitting and receiving transducers 40 mm in diameter (Figure 4).

In the set used, the transmitting transducer generated a 40 kHz ultrasonic wave. The range and accuracy were 32.64 to 39.98 µs and 0.02 µs, respectively. A special pH-neutral gel was used as a coupling agent. Each use of the defectoscope was preceded by calibration on a standard sample. All tests were performed at an ambient temperature of 24 ± 2 °C and relative humidity of about 60%.

The samples, between the successive test points, were stored in sealed containers in water at 24 ± 2 °C. Each time before ultrasonic testing, the samples were subjected to drying at 30 °C and forced air circulation conditions in a 110 dm^3^ Thermo Scientific Heraeus_®_ automatic oven for 22 h to remove the water present in the pores and cracks of the sample. The drying temperature does not negatively affect the hydration of the binders as it corresponds to the normal conditions of concrete exposure, whereas forced-air circulation speeds up the sample drying process. Using carborundum stone, impurities, outgoing cement paste, aggregate fragments, and dried coupling gel residues from previous tests were removed from the surface of the samples to which the ultrasonic transducers were applied. After the preparation of the sample surface before crack induction and immediately afterward, the dimensions of the samples were measured using a caliper with an accuracy of 0.02 mm and a digital measurement indicator.

The samples were placed, immobile, on a polyurethane mat, separating them from the ground during UPV measurements. The measurement was performed at least twice on the front surfaces of the sample to obtain a stable indication of the time of passage of the wave through the sample. Regardless of the number of measurements repeated on a given sample, each time, the amount of coupling agent dosed was similar, since it was previously verified that the amount of coupling agent influenced the results. The heads were positioned so that they did not extend beyond the edges of the sample. Since the dimensions of the sample were 40 × 40 mm, it allowed for a reproducible head position during all the measurements.

The analysis procedure for the test data, being an integral part of the presented method, is described below. Section 5, Section 6 and Section 7 present the results and discussion of the obtained results.

## 4. UPV Sensitivity to Sample Moisture

During the maturation period and after the deterioration process, samples were stored in water and were removed from the water each time for measurements. The moisture content of the tested samples had a significant influence on the UPV measurements [19], especially the incremental and relative results. Therefore, the influence of the moisture content of the samples on the UPV measurements was estimated. In the proposed method (Section 2), a constant drying time was proposed under the same conditions as those indicated by the preliminary results, ensuring the stability of the wave velocity measurements.

Tests were conducted on a separate set of 24 samples of four mortars: M5_0.3, M5_0.5, M20_0.3, and M20_0.5 (Table 2). The samples were prepared according to the procedures described in Section 2. Three samples with and without cracks were tested for each mortar. The test group of samples was selected to introduce a controlled variability of the material properties within the population, especially in terms of the tightness of the mortar microstructure. Before testing, the samples were stored in water for six months. After this time, the UPV and mass of the samples were determined immediately after their removal from the water (*d_0_*) and after *d_i_* = 1, 3, 6, 11, and 22 h of drying at 30 °C in an automatic oven with forced air circulation. The relative change in UPV and the relative change in mass, calculated from Equations (2) and (3), respectively, were used to evaluate the changes in the material due to drying:

(1) $$v_{d_{i\;}k}=L_{k}/T_{d_{i\;k}}$$

(2) $${\bar{∆v}}_{d_{i}}=\frac{1}{K}\overset{K}{\underset{k=1}{\sum }}\left(\left(v_{d_{i\;}k}-v_{0\;k}\right)/v_{0\;k}\right)\times 100\%$$

(3) $${\bar{∆m}}_{d_{i}}=\frac{1}{K}\overset{K}{\underset{k=1}{\sum }}\left(\left(m_{d_{i\;}k}-m_{0\;k}\right)/m_{0\;k}\right)\times 100\%$$

where$\;L_{k}$ is the distance traveled by ultrasonic waves (sample length) for sample number *k*;$\;T_{d_{i\;k}}$ is the time of passage of waves through the test material of sample *k* at time point *d_i_* during drying; *K* is the total number of samples of a given mortar, *k* = 1, …, *K*;$\;v_{d_{i\;}k}$ and $m_{d_{i\;}k}$ are the UPV and the mass of a sample *k* at the drying time point *d_i_*, respectively; and$\;v_{0\;k}$ and $m_{0\;k}$ are the UPV and the mass of sample *k* at time point *d_0_*, respectively.

**Figure 5:**
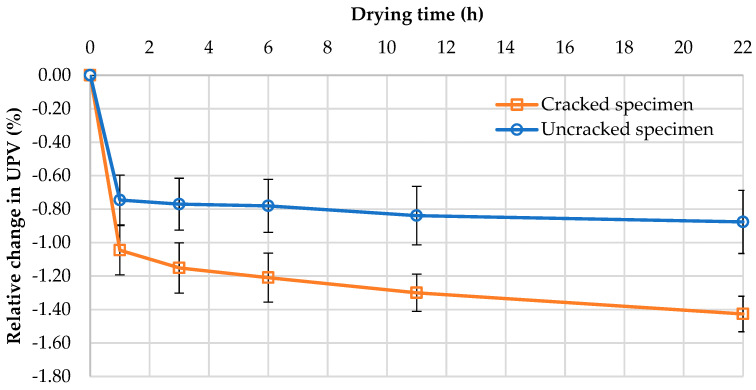
Relative change in the ultrasonic pulse velocity (∆v) of the mortar samples depending on the drying time; average values (according to Equation (2)); ranges ± standard deviation.

**Figure 6:**
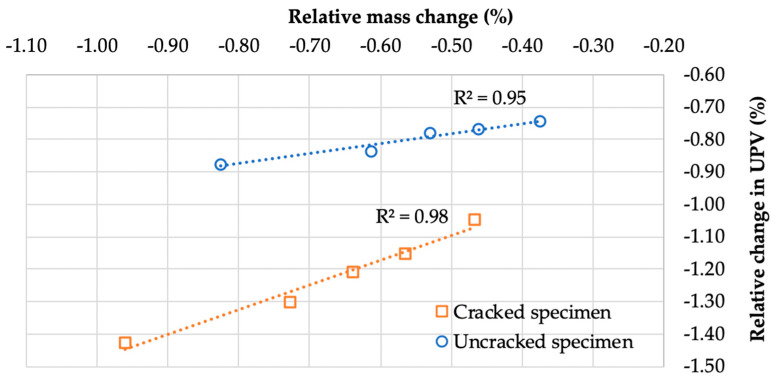
Relationship between the relative change in ultrasonic pulse velocity (∆v) and the relative change in mass $(\bar{∆m}$ ) of mortar samples (according to Equations (2) and (3), respectively) during drying.

Figure 5 shows the average relative changes in UPV $\bar{∆v}$ depending on the drying time; Figure 6 depicts the average changes in UPV depending on the average relative changes in the sample mass $\bar{∆m}$ resulting from water loss during drying.

According to the results in Figure 5, the UPV decreased with increasing drying time and decreasing moisture. After removing the samples from the water and drying for 22 h, the average relative decrease in UPV due to drying was 1.4% (63 m/s) for the samples with cracks and 0.9% (41 m/s) for the samples without cracks. The average relative decrease in mass related to the evaporation of water from the samples after 22 h of drying was about 1.0% for the samples with cracks and 0.8% for the samples without cracks. The further drying of the samples did not produce significant changes in UPV or mass; therefore, from a practical point of view, the suggested drying time is limited to the indicated 22 h. The results were also analyzed for each mortar separately. The changes in UPV and mass during drying were similar for mortars with the same w/b, with these changes being almost two times greater for the samples of mortars with w/b = 0.5 than the samples of mortars with w/b = 0.3. However, the presence of water in cracks more significantly affected the UPV and mass changes measured. The presence of water in the cracks in the mortar samples resulted in UPV and mass changes up to 2.4 times greater, during drying, than the changes in the uncracked samples.

As shown in Figure 5, samples with and without macrodefects differed in moisture content due to water accumulation in the additional volume of cracks. UPV was strongly dependent on the water content of the samples (Figure 6), and the UPV in water was nearly four times higher than that in air. Therefore, water remaining in pores and cracks will reduce the differences in microstructure between cracked and uncracked samples, thus affecting the accuracy of UPV measurements. The proposed drying condition improves the reliability of the data obtained in the evaluation of the self-sealing process and reduces the dispersion of the UPV measurements. The coefficient of variation of UPV within the experimental groups of samples was 0.5% on average.

## 5. Absolute and Relative Changes in UPV

To examine the changes in the ultrasonic pulse velocity of the mortar samples during the self-sealing treatment, 792 measurements (double measurement on a total of six samples for each of six mortars at 11 time points) were recorded following the procedures described in Section 3. The ultrasonic pulse velocity $v_{t_{i}\;k}$ at time point *t_i_* for sample *k* was calculated as the sample length over the wave passage time (Equation (4)). The average ultrasonic pulse velocities ${\bar{v}}_{t_{i}}$ for all samples of a given mortar were calculated according to Equation (5). To describe the changes in the ultrasonic wave velocity at time point *t_i_* due to the induction of cracks and subsequent self-sealing, a measure of the relative change in UPV, $∆v_{t_{i}}$, was used with reference to UPV before cracking at time point *t_0_,* according to Equation (6):

(4) $$v_{t_{i}\;k}=L_{k}/T_{t_{i}\;k}$$

(5) $${\bar{v}}_{t_{i}}=\frac{1}{K}\overset{K}{\underset{k=1}{\sum }}v_{t_{i\;}k}$$

(6) $$∆v_{t_{i}}=\left({\bar{v}}_{t_{i\;}}-{\bar{v}}_{0}\right)/{\bar{v}}_{0}\times 100\%$$

where$\;L_{k}$ is the distance traveled by ultrasonic waves (sample length) for sample number *k*;$\;T_{\;t_{i\;k}}$ is the time of passage of waves through the test material at time point *t_i_* for sample *k*; *K* is the total number of samples of a given mortar, *k* = 1, …, *K*;$\;{\bar{v}}_{t_{i}}$ is the average ultrasonic pulse velocity for a given mortar at time point *t_i_*; and$\;{\bar{v}}_{0}$ is the average ultrasonic pulse velocity in a group of uncracked samples of a given mortar at time point *t_0_*.

**Figure 7:**
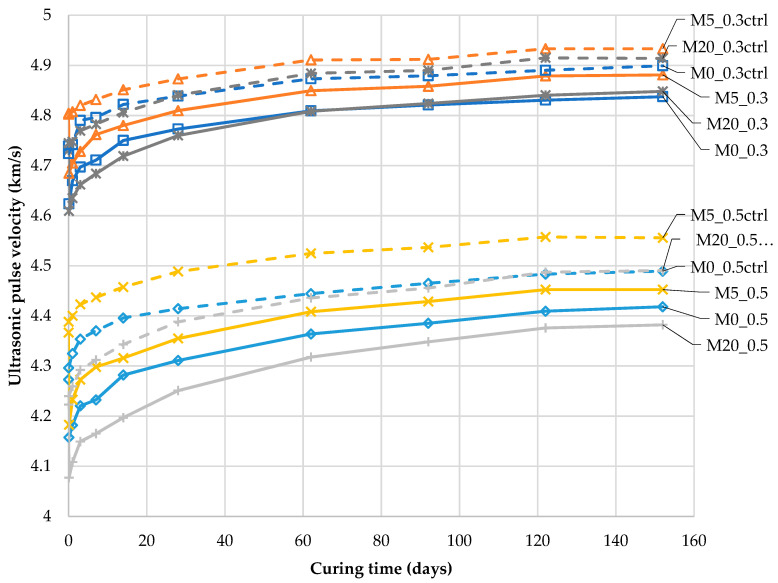
Ultrasonic pulse velocity ($\bar{v}$) in cracked mortar samples during self-sealing time, and ultrasonic pulse velocity ($\bar{v}$ ) in uncracked mortar samples (“-ctrl”) during further hydration after 28 days of maturation (according to Equation (5)).

Figure 7 presents graphs of the average UPV $\bar{v}$ (according to Equation (5)) over time in the samples in which cracks were induced on the 28th day of maturation. The decrease in the UPV caused by the creation of cracking space (at time point *t_0_*) is illustrated in the graphs by two points corresponding to the UPV immediately before (${\bar{v}}_{0}$) and after the defect induction (${\bar{v}}_{ind}$). In Figure 7, the UPVs specified for the control samples without intentionally introduced cracks are shown by dotted lines. This data presentation system corresponds to the schematic representation in Figure 1 of changes in the composite from the moment of material degradation in relation to changes in the material during undisturbed maturation in undamaged material. The division of the mortar samples into two groups according to their water-to-binder ratio is clear.

**Figure 8:**
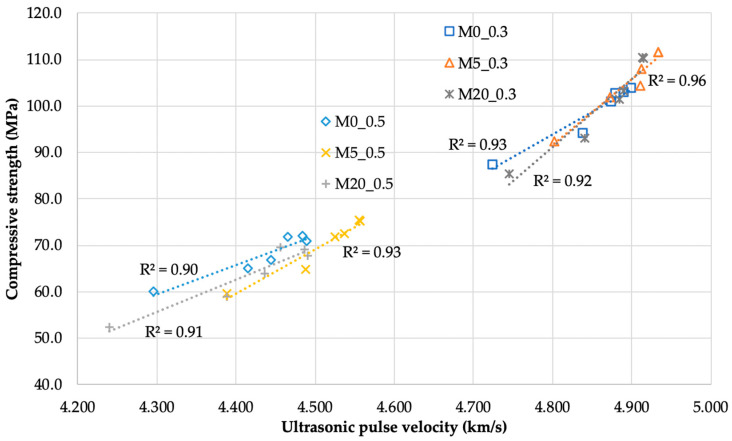
Relationship between the compressive strength of the mortars and ultrasonic pulse velocity after different maturation times.

A clear division of the compressive strength of the mortars into two ranges, related to the two water-to-binder ratios, is visible in Figure 8.

Figure 8 shows the relationship between the UPV and the compressive strength specified for individual mortars on an additional set of samples. The measurements were recorded at different time points in the test (Section 2), so it was possible to observe that an increase in the strength of the mortars due to maturation and increased material density also increased UPV. There is a clear linear relationship between these parameters. A 20% substitution of cement with siliceous fly ash significantly reduced the initial binder activity, which manifested in 2.5% and 14.6% lower compressive strength of the M20_0.3 and M20_0.5 mortars than the M0_0.3 and M0_0.5 mortars, respectively. After 152 days of curing only for the M20_0.5 mortar, the compressive strength was lower by 4.7% than that of the M0_0.5 mortar. In the remaining cases, the addition of siliceous fly ash resulted in an increase of 5.9% to 8.4% in strength in comparison to mortars without this addition, with the largest increase being observed for the M5_0.3 mortar.

**Figure 9:**
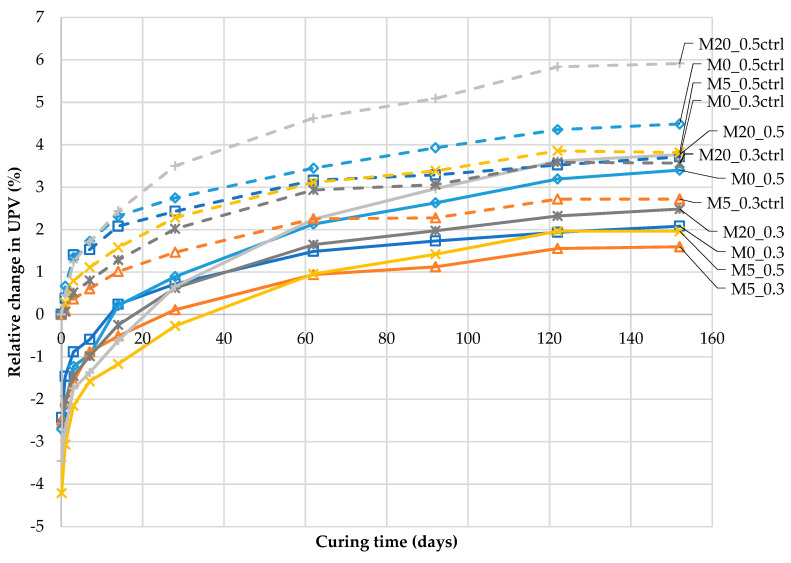
Relative changes in the UPV (∆v) in cracked mortar samples during self-sealing, and relative changes in the UPV (∆v ) in uncracked mortar samples (“-ctrl”) during further hydration after 28 days of maturation (according to Equation (6)).

Figure 9 presents the data from Figure 7 as relative values, according to Equation (6).

As shown in Figure 9, as a result of an occurring crack, the UPV decreased by a maximum of 4.2% (190 m/s) compared to the UPV prior to deterioration (${\bar{v}}_{0}$). After 152 days of curing, the UPV increased by a maximum of 5.9% (267 m/s) compared to the initial UPV (${\bar{v}}_{0}$) in the control samples during further hydration. In this context, the 1.4% influence of the moisture content on the obtained ultrasonic results (as shown in Section 4) is a significant source of measurement uncertainty. Disregarding this factor may lead to a large scattering of UPV, specifically for those observed at subsequent time points of examination. For the differences found for cracked and uncracked samples, as depicted in Figure 5, the abandonment of drying would cause systematic error due to the larger water content in the cracked samples.

The data expressed as relative values (Figure 9) are dependent on the UPV at time point *t*_0_ before cracking (${\bar{v}}_{0}$), which is the divisor in Equation (6). Therefore, for mortars with initially low UPV (such as mortars with w/b = 0.5, Figure 7), the recorded relative changes in the UPV over time are higher than those for mortars with lower w/b. This complicates the appropriate assessment of the changes caused by self-sealing.

**Figure 10:**
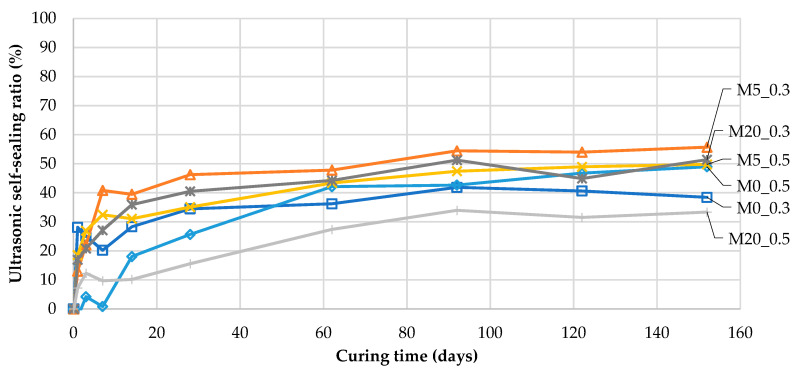
Ultrasonic self-sealing ratio (USR) during curing time (according to Equation (7)).

Concerning the changes in the properties of cement-based composites over time described in Section 1, in general, the changes in the tested parameters during self-sealing were influenced by further hydration of the cement and a possible pozzolanic reaction and/or the latent hydraulic properties of supplementary cementitious materials and pure self-sealing. A set of control samples without cracks was used to assess natural changes in the UPV during further hydration of the composite. The changes in UPV as a result of pure self-sealing were determined according Equation (7) and are presented in Figure 10. This is hereafter referred to as the ultrasonic self-sealing ratio, $USR_{t_{i}}$:

(7) $$USR_{t_{i}}=\left(\left(\left({\bar{v}}_{t_{i}}-{\bar{v}}_{ind}\right)-\left({\bar{v}}_{t_{i}}^{ctrl}-{\bar{v}}_{0}^{ctrl}\right)\right)/\left(\;{\bar{v}}_{0}^{ctrl}-{\bar{v}}_{ind}\right)\right)\times 100\%$$

where$\;{\bar{v}}_{t_{i}}^{ctrl}$ and ${\bar{v}}_{0}^{ctrl}$ are the average ultrasonic pulse velocities for a group of control samples (without cracks) of a given mortar at the time points *t_i_* and *t_0_*, respectively;$\;{\bar{v}}_{t_{i}}$ is the average ultrasonic pulse velocity for a group of samples (with cracks) of a given mortar at a point in time *t_i_;* and$\;{\bar{v}}_{ind}$ is the average ultrasonic pulse velocity for a group of samples of a given mortar for the state immediately after cracks were induced. Łukowski and Adamczewski [14] proposed a “self-repair degree” measure based on compressive strength test results, defined with an analogous mathematical formula.

USR is the measure of pure self-sealing. It allows the estimation of the effective degree of filling of the defect volume precipitated in the process of self-sealing with the material, as well as the density changes near the defect surface. However, the range of structure compaction is strongly limited, especially in the case of composites with high strength and dense microstructure. These changes would not occur in the absence of a defect, so they can be classified together as pure self-sealing.

In Figure 10, it can be seen that when the ultrasonic self-sealing ratio is zero, it is related to the initial state directly after cracking without any changes inside the cracks. When this parameter is equal to 100%, the defect volume is completely filled with an equivalent material characterized by the same UPV as the sample material. The obtained values of the ultrasonic self-sealing ratio after 152 days of curing indicated that the defects were partially filled with equivalent material in the range of 33% to 57%, with the highest filling being observed for the M5_0.3 mortar samples. Since the UPV measurements were used as an indicator, the values do not describe exact volumetric changes in the self-sealing products’ formation but rather their effect on the properties of the material. The self-sealing process depends on the amount of products formed, their distribution throughout the material, the size of the grains, etc. UPV measurements estimate the net effect of the self-sealing process on the properties of the material. The method allows for tracing those changes for the same samples throughout the investigated period.

To examine the relationship between the ultrasonic self-sealing ratio (USR) and the reduction in crack width at the outer surface of the samples, computer processing and analysis of the samples’ surface image was used [34] with a total of 25,080 cracks’ width measurements (an average of 418 measurements for each of six mortars at 10 time points). The average crack widths at any time point *t_i_* and relative changes in crack widths ($∆cw_{t_{i}}$) in relation to the initial crack widths were calculated according to Equations (8) and (9), respectively:

(8) $${\bar{cw}}_{t_{i}}=\frac{1}{M}\overset{M}{\underset{m=1}{\sum }}cw_{t_{i\;}m}$$

(9) $$∆cw_{t_{i}}=\left({\bar{cw}}_{ind}-{\bar{cw}}_{t_{i\;}}\right)/{\bar{cw}}_{ind}\times 100\%$$

where $cw_{\;t_{i\;m}}$ is the crack width measurement *m* at time point *t_i_*; *M* is the total number of crack width measurements of a given mortar, *m* = 1, …, *M*;$\;{\bar{cw}}_{t_{i}}$ is the average width of the cracks in samples of a given mortar at time point *t_i_*; and$\;{\bar{cw}}_{ind}$ is the average width of the cracks in samples of a given mortar for the state immediately after cracks were induced.

**Figure 11:**
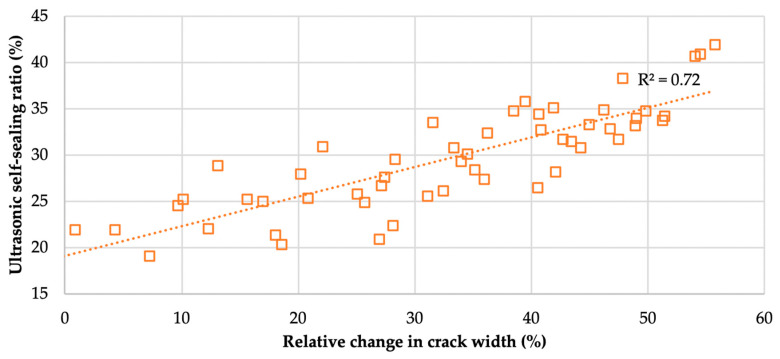
Relationship between the relative change in crack width (according to Equation (9)) and ultrasonic self-sealing ratio (USR) in all mortar samples after different curing times.

The linear relationship between the parameters describing the internal filling of cracks and the relative change (reduction) in crack width does not necessarily have to occur in all self-sealing experiment results. Despite the chemical and microstructural substrate being the same, the phenomena of the internal filling of cracks and reduction of crack width on the external surface may not occur proportionally. The accumulation of newly formed material in the area of the cracks near the external surface of the samples may lead to the sealing of only the mouth of the crack without significant changes inside the tested material [17]. However, as shown in Figure 11, in the experiment under consideration, such a relation is apparent and significant.

## 6. Effects of Fly Ash on Self-Sealing

**Figure 12:**
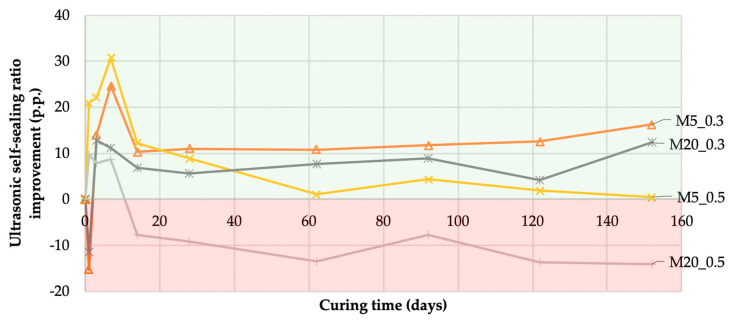
Difference in the self-sealing ratio between mortars with and without siliceous fly ash (USRI) during curing time (according to Equation (10)). Note: The green and red ranges in the graph indicate the positive and negative effects of mineral additions on the efficiency of self-sealing, respectively.

The abovementioned relative changes in UPV allowed the analysis of the impact of the mineral additions on self-sealing. The differences in the self-sealing ratios between mortars with fly ash and their equivalents without fly ash, i.e., mortars M0_0.3 and M0_0.5, were determined as ultrasonic self-sealing ratio improvement, $USRI_{t_{i}}$, using Equation (10) and are presented in Figure 12:

(10) $$USRI_{t_{i}}=USR_{t_{i}}^{Mp\_q}-USR_{t_{i}}^{M0\_q}$$

where$\;USR_{t_{i}}^{Mp\_q}$ is the ultrasonic self-sealing ratio at time point *t_i_* for mortar samples *Mp_q*$,\;USR_{t_{i}}^{M0\_q}$ is the ultrasonic self-sealing ratio at time point *t_i_* for samples of the reference mortar *M0_q* created without mineral addition, and p is the substitution of cement with fly ash. In the present work, the substitutions were 0, 5, and 20, meaning 0%, 5%, and 20% substitution of cement by fly ash, respectively; q is the water-to-binder ratio, equal to 0.3 or 0.5.

The results of the data analysis indicated that, in most cases, the incorporation of siliceous fly ash increased the self-sealing capacity. After the first day of self-sealing, all mortars with w/b = 0.3 and fly ash were characterized by lower wave velocities, resulting in self-sealing higher by 11 to 15 percentage points (p.p.) in relation to the M0_0.3 mortar. This indicated an initial slowdown in the self-sealing process due to the mineral addition used, which is less active than Portland cement. This phenomenon was described previously [39], among others. After seven days of self-sealing, all the mortars with fly ash had a higher wave velocity resulting from self-sealing than the mortars without a cement substitute. After 152 days of self-sealing, in the group of mortars with w/b = 0.3, the highest increase in the self-sealing ratio of 16 p.p. compared to the M0_0.3 mortar was observed for the M5_0.3 mortar. In the analogous comparison, an increase in the self-sealing ratio by 12 p.p. was observed for the M20_0.3 mortar. However, in the group of mortars with w/b = 0.5, only M5_0.5 showed an increase in the self-sealing ratio by 1.0 p.p. M20_0.5 showed a 16 p.p. lower self-sealing ratio than M0_0.5. This decrease was related to the low binder activity in the M20_0.5 mortar compared to the cement activity in the M0_0.5 mortar. This was confirmed by the results of the mortar compression strength test, which was used to measure binder activity, as described in Section 5. This suggested that the process of self-sealing and strength development has a similar substrate.

## 7. Dynamics of UPV Changes Over Time

As a measure of the dynamics of changes in the UPV over time during the course of self-sealing, the rate of relative UPV change, $USRC_{t_{i}}$ (hereafter referred to as the ultrasonic self-sealing rate of change), was calculated for the time point *t_i_* using the following:

(11) $$USRC_{t_{i}}=\left(USR_{t_{i}}-USR_{t_{i-1}}\right)/\left(t_{i}-t_{i-1}\right)$$

where$\;USR_{t_{i}}\;$and $USR_{t_{i-1}}$ are the ultrasonic self-sealing ratio at time points *t_i_* and *t_i-1_*, respectively, and *i* ≥ 1.

**Figure 13:**
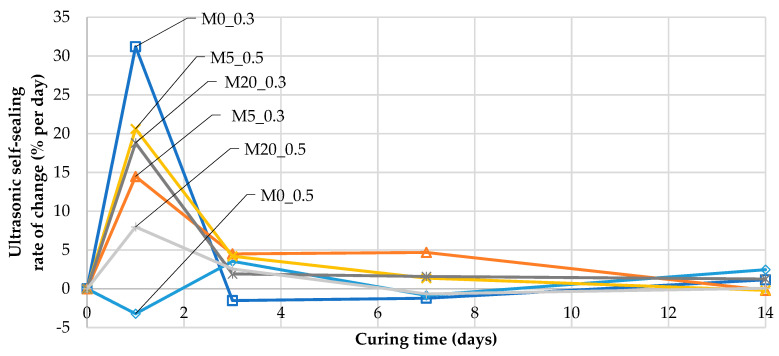
The changes in the ultrasonic self-sealing rate of change (USRC) over time in mortar samples during the first 14 days of curing after the induction of cracks (according to Equation (11)).

Figure 13 shows the values of the ultrasonic self-sealing rate for the mortar samples, indicating the percentages of the initial UPV recovered in one day of curing after the induction of cracks and how these values changed over time.

For the M0_0.5 mortar (without fly ash, w/b = 0.5), during the first days of self-sealing, the changes in UPV in the samples with cracks occurred significantly more slowly than in the samples without them. For the remaining mortars, a characteristic feature was a maximum ultrasonic self-sealing rate of 8% to 31% per day after the first day of curing. The highest ultrasonic self-sealing rate after that time was found for M0_0.3 (31% per day) without supplementary cementitious material, but after three days of self-sealing, the ultrasonic self-sealing rate for this mortar decreased to under 0% per day. Another mortar showing a high ultrasonic self-sealing rate after the first day of curing was M5_0.5 (21% per day). After three days of curing, the highest ultrasonic self-sealing rate was observed for mortars M5_0.3 (5% per day) and M5_0.5 (4% per day). After 14 days of self-sealing, the average rate slowed to a maximum level of 2% per day. After 152 days of curing, the highest ultrasonic self-sealing rate was 0.2% per day in M20_0.3. For comparison, M0_0.3, without fly ash, had a rate of change nearly equal to 0% per day. For mortars with w/b = 0.5, the use of fly ash as a cement substitute decreased the rate of changes due to self-sealing in comparison with those in M0_0.5, composed of neat cement.

## 8. Optical and SEM Observation

**Figure 14:**
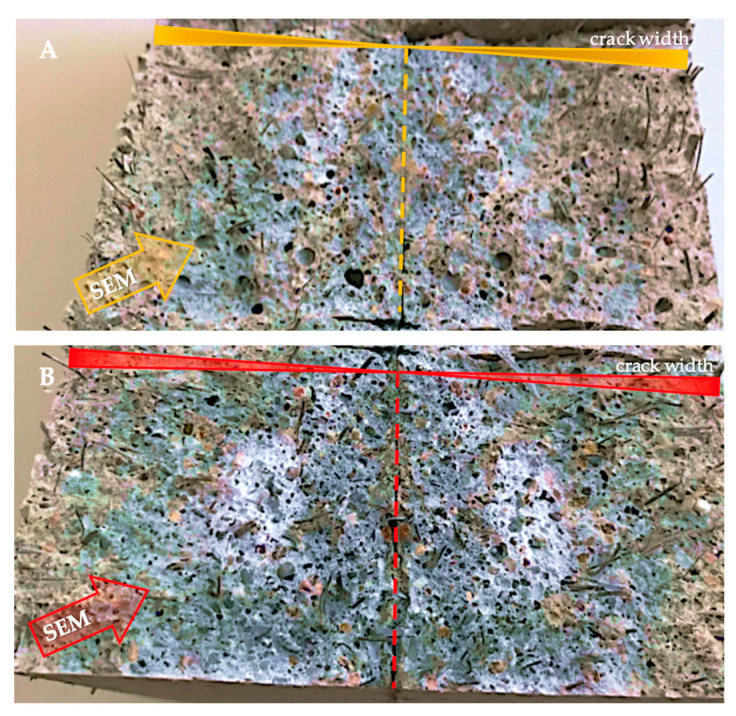
Images of the cracks’ surface after separating samples of the (**A**) M0_0.3 and (**B**) M5_0.3 mortars into two parts, together with the newly created self-sealing light-colored material. The dashed line distinguishes the area with 0 crack width; crack width bars indicate the direction of the crack width increase from 0 to the maximum width; the areas subjected to SEM observations are marked.

**Figure 15:**
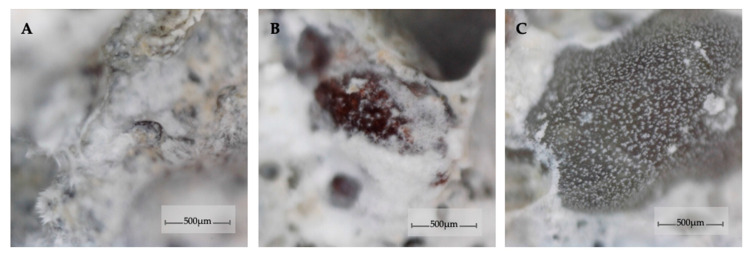
The concentration of self-sealing products observed at the fractures in M0_0.3 mortar samples appearing on the surface of the cracks in the area of (**A**) pores, (**B**) cement paste, and (**C**) aggregate grain.

To examine the material newly formed inside the cracks after 152 days of curing, the samples were separated into two halves to expose the surface of the cracks. Pictures of the fracture surfaces of the M0_0.3 and M5_0.3 mortar samples are shown in Figure 14. This picture shows the varying amount of the newly formed material in light color depending on the fly ash content. Differences in the newly formed material were also visible between samples of other mortars. According to the analysis of the crack width distribution along the cracks on the outer surface of the samples, the highest concentration of newly formed material at the fracture site appeared mostly in crack areas of 100 to 200 µm, and the concentration of the material decreased with an increase in the crack width. In cracks less than 100 µm wide, a layer of newly formed material was observed, but the amount (and intensity of light in the pictures) was lower due to the limitation of the crack volume in this area. The picture also shows the fracture area that was used for SEM observation with EDS analysis. Figure 15 depicts 300× magnification images from an optical microscope with a digital image recording showing the morphology of the self-sealing products on the surface of the mortar sample fractures. Clear differences were observed in the concentration of self-sealing products in the cement paste and aggregate grain area.

**Figure 16:**
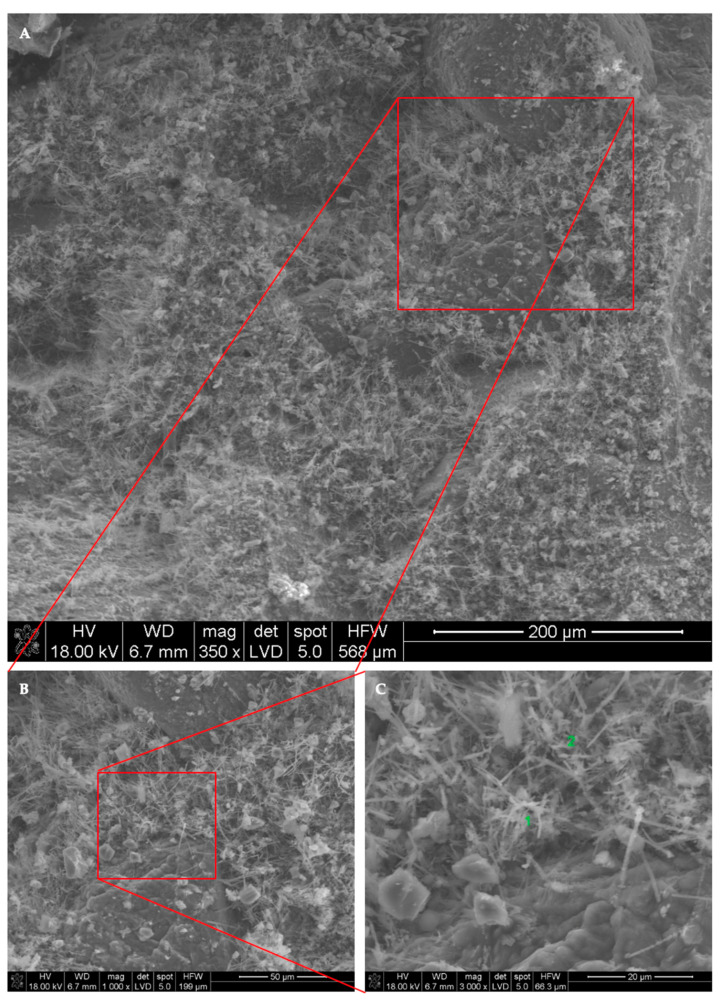
Typical SEM image of crack surfaces in M0_0.3 mortar sample (**A**) in the area of the cement paste and aggregate grains and (**B**,**C**) with a magnification of micro-areas during EDS analysis.

**Figure 17:**
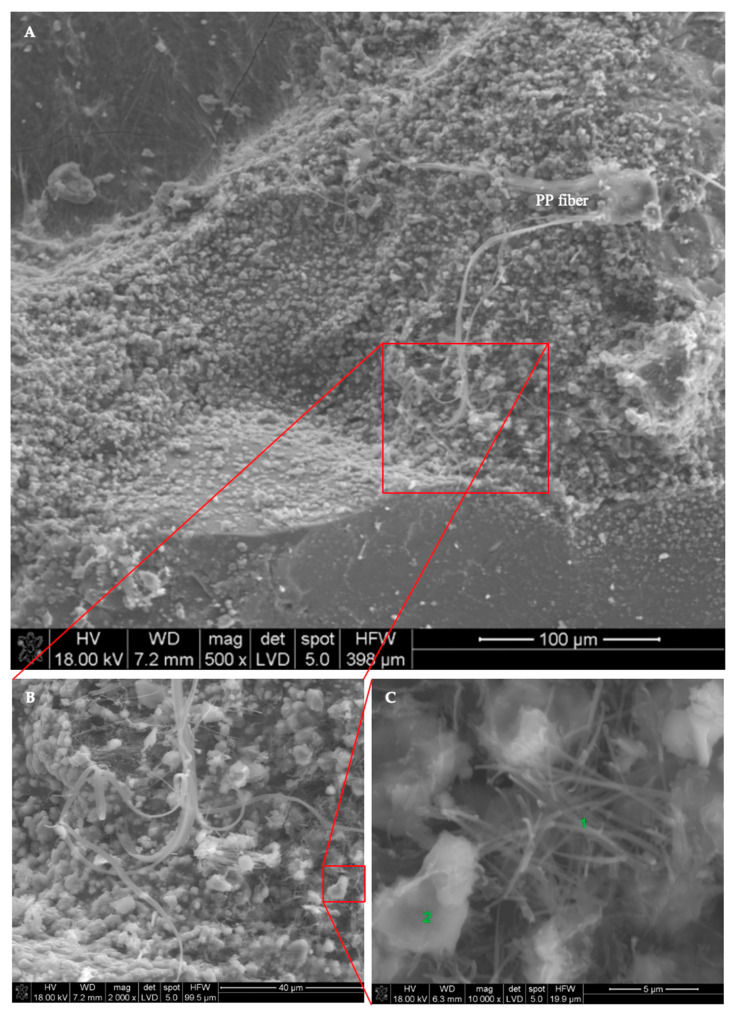
Typical SEM image of crack surfaces in M5_0.3 mortar sample (**A**) in the area of the cement paste and aggregate grains and (**B**,**C**) with a magnification of micro-areas during EDS analysis.

**Figure 18:**
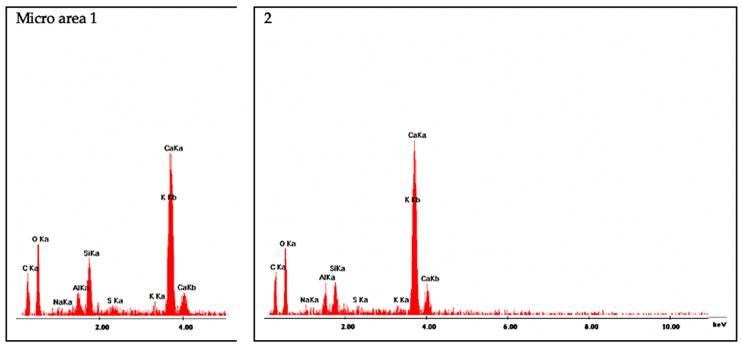
EDS spectra for micro-areas on M0_0.3 sample labeled numerically in Figure 16C (micro-areas 1 and 2: the form of fibrous C–S–H phase with high calcium content).

**Figure 19:**
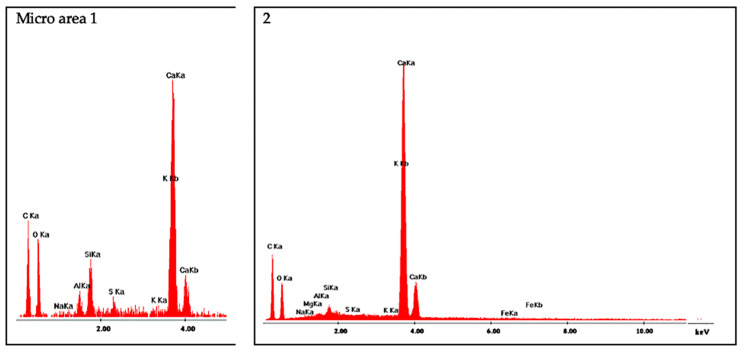
EDS spectra for micro-areas on M5_0.3 sample labeled numerically in Figure 17C (micro-area 1: C–S–H phase with high calcium content, 2: calcium carbonate).

The morphology and distribution of self-sealing products on the surface of cracks in the tested M0_0.3 and M5_0.3 mortar samples are shown in Figure 16 and Figure 17, respectively. EDS analysis was carried out on the micro-areas of both samples, the results of which are shown in the form of spectra in Figure 18 and Figure 19, respectively.

Microstructure examinations with SEM accompanied by EDS showed that the main self-sealing product formed in the crack area of the cement-based mortar samples was a fibrous C–S–H phase with high calcium content, forming a porous layer together with fine calcium carbonate particles. For the mortar samples modified with siliceous fly ash, the main product of self-sealing was calcium carbonate, which formed a compact layer supplemented with a fibrous C–S–H phase with high calcium content. The most intensive crack fillings covering the largest area of the fractures were observed for the M0_0.3 mortar samples. As such, it was concluded that for self-sealing performance estimated by the proposed measures based on UPV measurements, the concentration of the newly formed materials in the cracks and the nature of these products are important. For intensive crack filling with the fibrous C–S–H phase, the resulting change in UPV may be less noticeable than that with densely packed calcium carbonate. The degree of the restoration of the original compactness of the composite (measured using UPV and expressed in USR and USRI values), lost due to the formation of cracks, may be greater in the case of filling with calcium carbonate than that with the fibrous C–S–H phase. Despite the presence of the C–S–H phase in the defect volume, we cannot comment on the recovery of the strength of the composite because this was outside the scope of this study. Based on the literature data, it can be assumed that the appearance of a large amount of calcium carbonate filling the cracks may indicate a limited recovery of strength properties in the composites tested [40].

The above observations are consistent with the conclusions from the pure self-sealing studies estimated based on the ultrasonic self-sealing ratio (USR), which omits the magnitude of the UPV changes caused by subsequent binder hydration. This provides further justification for the adopted test methodology.

## 9. Conclusions

The objective of this study was to develop a method for conducting tests and analyses of ultrasonic measurements used for assessing the progress of the self-sealing process in concrete. To eliminate the influence of progressive hydration on the measurements, to assess the influence of the moisture content of ultrasound tested samples, and to quantitatively describe the changes in the self-sealing and the stimulation of this process with mineral additives, special procedures for sample preparation, specific test conditions, and measurement result processing were applied. The following were concluded:

1. Uncontrolled and random moisture content in samples, both in the material and in the crack volume, may significantly bias the results of the ultrasonic pulse velocity measurements and the conclusions upon which they are based. The procedure for drying samples under forced air circulation for 22 h at 30 °C does not adversely affect the binder hydration process, instead providing stable, reproducible moisture conditions for ultrasonic velocity measurements. The proposed sample preparation procedure, test plan, and measurement method capture the clear trend in the UPV-based results over time with slight deviations from the general trend.
2. Based on the results obtained, new parameters quantitatively describing the self-sealing process were proposed: the USR (ultrasonic self-sealing ratio) quantifies the effective degree of the filling of the cracks with self-sealing products, which allows the estimation of the restoration of the properties of the material from the UPV point of view; the USRI (ultrasonic self-sealing ratio improvement) allows the quantification of the influence of any mineral additive incorporated into the material on its self-sealing ability, which clearly distinguishes between the positive and negative influence of mineral additives on self-sealing. It is also easy to analyze the dynamics of the abovementioned parameter changes, since the examinations are performed on the same set of samples at any time point, and examination is non-destructive for the samples. The dynamics of the self-sealing process can be quantitatively recognized, and the impact of various factors (mineral additive presence, water-to-binder ratio, curing scheme, etc.) on the efficiency of self-sealing changes over time can be assessed using the USRC (ultrasonic self-sealing rate of change).
3. Based on the results of crack width measurements obtained by computer processing and the analysis of surface images of the samples, it was shown that there is a significant linear relationship between the crack filling inside samples, as quantified by USR, and the magnitude of the crack width changes (reduction) at the outer surfaces of the samples during the self-sealing process.
4. For mortars with siliceous fly ash with a water-to-binder ratio of 0.3, a significant increase in the amount of change occurring inside the material was observed due to self-sealing in relation to the comparative mortar without this addition. However, for mortars with a water-to-binder ratio of 0.5, the effect of the modification of the composite with a high amount of siliceous fly ash in this range was negative. For mortars with a more compact microstructure and modified with the mineral addition, the initial dynamics of the self-healing process measured by ultrasonic self-sealing rate of change (USRC) were slower than those for analogous mortars where the only binder was Portland cement. This may be caused by the low reactivity of the fly ash compared to that of cement, which was confirmed by compressive strength tests of the mortars. The experimental results suggest that the optimum substitution of cement by siliceous fly ash from the perspective of the efficiency of self-sealing is between 5% and 20% by mass.
5. Based on SEM observations with EDS analysis, qualitative differences between samples of different mortars in terms of the newly formed materials inside the cracks were confirmed. In the mortar samples without mineral addition, the self-sealing products were mainly C–S–H phase with high calcium content; for the mortars with siliceous fly ash, they were mainly calcium carbonate. It was observed that the degree of filling, estimated by measures based on UPV measurements, is influenced by the density of newly formed products in addition to the material concentration in the cracks. Observations of the microstructure confirmed the results of the ultrasonic measurements.

## Figures and Tables

**Table 1:** Chemical composition of the binders used.

Component	CEM I 42.5R (%)	Siliceous Fly Ash (%)
SiO_2_	17.90	46.92
Al_2_O_3_	5.60	28.54
Fe_2_O_3_	2.61	7.23
CaO	63.11	3.39
MgO	1.17	2.31
TiO_2_	0.36	1.32
SO_3_	4.02	1.87
Na_2_O	0.34	4.83
K_2_O	0.78	2.08
P_2_O_5_	0.13	0.31
Cl	0.09	0.02
other	0.25	0.45
Loss on ignition (1 h at 1000 °C)	3.66	0.72
CaO_free_	1.49	0.30
Na_2_O_eq_^1^	0.88	6.24

^1^ Na_2_O_eq_ = Na_2_O + 0.658 K_2_O.

**Table 2:** Mix proportions of mortars. Note: *Mp_q,* mortar with *p*% substitution of cement with siliceous fly ash and w/b = *q*; SP, CHRYSO_®_ Optima 185 superplasticizer; PP, Astra Polyex Mesh 2000_®_ polypropylene fibers; m_b_, total mass of binder; w/b, water-to-binder ratio.

Mortar *Mp_q*	CEM I 42.5R (g)	Siliceous Fly Ash (g)	CEN-Standard Sand (g)	Water (g)	SP (g)	SP (%m_b_)	PP (g)	w/b
M0_0.3	550.0	0.0	1350.0	160.8	5.50	1.00	15.63	0.30
M0_0.5	550.0	0.0		274.5	0.66	0.12	17.61	0.50
M5_0.3	522.5	27.5		157.7	9.63	1.75	15.70	0.30
M5_0.5	522.5	27.5		274.0	1.38	0.25	17.68	0.50
M20_0.3	440.0	110.0		149.4	20.63	3.75	15.93	0.30
M20_0.5	440.0	110.0		272.9	2.75	050	17.87	0.50
